# Characterization of the complete chloroplast genome of *Citrus hongheensis*, a key protected wild plant in Yunnan province of China

**DOI:** 10.1080/23802359.2020.1825137

**Published:** 2020-10-07

**Authors:** Zi-hao Zhang, Chun-rui Long, Yan Jiang, Xue-jun Bei, Shaohua Wang

**Affiliations:** a Institute of Tropical and Subtropical Cash Crops, Yunnan Academy of Agricultural Sciences, Baoshan, PR China; b College of Horticulture, Fujian Agriculture and Forestry University, Fuzhou, PR China; c Institute of Dehong Tropical Agriculture Research Institute of Yunnan, Ruili, PR China; d College of Biology & Pharmacy, Yulin Normal University, Yulin, PR China

**Keywords:** *Citrus hongheensis*, chloroplast genome, phylogenetic analysis

## Abstract

*Citrus hongheensis* is a key protected wild plant endemic to the Honghe river region in southeastern Yunnan, China. In the present study, its chloroplast genome was successfully assembled and annotated based on the Illumina Hiseq-2500 whole genome re-sequencing data. The chloroplast genome is 160,275 bp in size. Its large single copy region, small single copy region and inverted repeat region is 87,886 bp, 18,387 bp and 27,001 bp, respectively. Totally, 114 unique genes, including 80 protein-coding genes, 30 tRNAs and 4 rRNAs, were identified from the *C*. *hongheensis* chloroplast genome. According to the phylogenetic analysis result, the relationship between the chloroplast genome of *C. hongheensis* and *C*. *maxima* was found to be the closest.

*Citrus hongheensis*, named as ‘Honghe Dayicheng’ or ‘Honghecheng’ in China for its original habitat and its long wing leaf, is endemic to the Honghe river region in southeastern Yunnan, China (Ye et al. 1976; Sheng et al. 1997; Yang et al. 2010). The height of *C. hongheensis* tree can be higher than 10 m. Its young leaves can be used as flavoring and its fruits can be used as Chinese herbal medicine. Most of the discovered *C. hongheensis* trees are mature trees, indicating that its hard reproduction and general decline in natural condition (Yang et al. 2010). Moreover, it has been listed as ‘Third Grade’ key protected wild plant in Yunnan province of China (Zhou 2010). Current researches on *C. hongheensis* are mainly focused on its morphological characteristics (Huang 1997), evolution and genetic variation (Yang et al. 2010) and so on. In our present study, we assembled and annotated the complete chloroplast genome using the whole genome re-sequencing data of *C. hongheensis* and analyzed its relationship with several *Citrus* species from chloroplast genome level.

The specimen of *C. hongheensis* was isolated from Puyi village, Yangjie town, Yuanjiang county, Yunnan province, China (23°24′23.40″N; 102°03′50.35″E) and samples were deposited at Institute of Tropical and Subtropical Cash Crops, Yunnan Academy of Agricultural Sciences. By using the CTAB method (Xie et al. 2020), total leaf genomic DNA of *C. hongheensis* was extracted. And DNA was stored at the Institute of Tropical and Subtropical Cash Crops, Yunnan Academy of Agricultural Sciences (No. HHDYC01). The whole genomic DNA re-sequencing was performed on the Illumina Hiseq-2500 platform to generate 125 bp pair end reads (BIG, Shenzhen, CA, CHN). Totally, we obtained about 8 G high quality clean reads, which were aligned to chloroplast genomes of *Citrus limon* (KY085897.1), *C. platymamma* (NC_030194.4), *C. sinensis* (DQ864733.1), *C. maxima* (NC_034290.1), *C. aurantiifolia* (KJ865401.1) *C. depressa* (LC147381.1) and *C. reticulata* (NC_034671.1), assembled into contigs using CLC Genomics Workbench v8.0 (CLC Bio, Aarhus, Denmark), and then annotated using DOGMA (Wyman et al. 2004) and Geneious (Kearse et al. 2012). The annotated chloroplast genome of *C. hongheensis* has been deposited in Genbank with the accession number MT880607.

The complete chloroplast genome of *C. hongheensis* is 1,60,275 bp in size, containing a large single copy region of 87,886 bp, a small single copy region of 18,387 bp, and a pair of inverted repeat regions of 27,001 bp. Sequence annotation identified 114 unique genes from the *C*. *ichangensis* chloroplast genome, including 80 protein-coding genes, 30 tRNA genes and 4 rRNA genes. Among them, nine of the 80 protein coding genes (i.e. *ndhB*, *rpl2*, *rpl22*, *rpl23*, *rps7*, *rps12*, *rps19*, *ycf2* and *ycf15*), seven of the 30 tRNA genes (i.e. *trnA-UGC*, *trnI-CAU, trnI-GAU*, *trnL-CAA*, *trnN-GUU*, *trnR-ACG* and *trnV-GAC*) and all the 4 rRNA genes (*rrn4.5*, *rrn5*, *rrn16* and *rrn23* rRNAs) occur in double copies, and others occur in a single copy. The overall nucleotide composition of the chloroplast genome is: 30.47% A, 31.06% T, 19.60% C, and 18.86% G, with the total GC content of 38.47%.

By using the complete chloroplast genomes of *C. hongheensis*, 20 plant species from Rutaceae family and *Ailanthus altissima* (as outgroup) (Xu et al. 2019), a maximum-likelihood phylogenetic tree was constructed to reveal its phylogenetic relationship with *Citrus* species from the chloroplast genome level. Results showed that *C. hongheensis* is close related to the Citrus species and the relationship between the chloroplast genome of *C. hongheensis* and *C. maxima* was the closest, which was consistent with the results reported by Wang and Jiang (2010) and Li et al. (2015) (Figure 1). The *C. hongheensis* complete chloroplast genome reported in this study can provide basis for the future origin and evolution researches of *Citrus* species.

**Figure 1.**
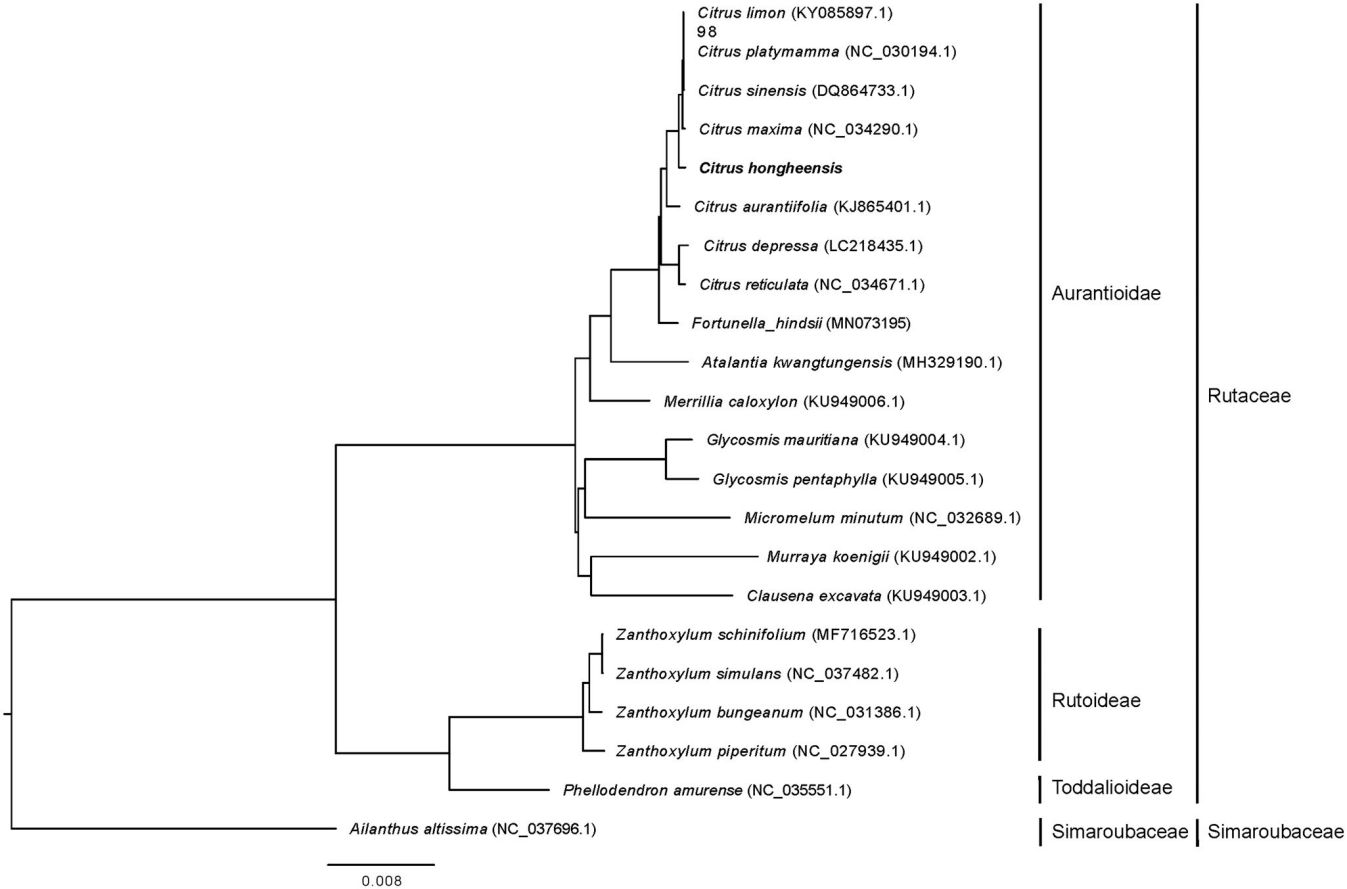
The maximum likelihood phylogenetic tree constructed using the complete chloroplast genome sequences of *C*. *hongheensis* and 20 Rutaceae species.

## Data Availability

The data that support the findings of this study is openly available in SRA at [http://www.ncbi.nlm.nih.gov/bioproject/658652] and in Genbank at [https://www.ncbi.nlm.nih.gov/genbank/] (accession number MT880607).
